# Rehabilitation to improve gaze and postural stability in people with multiple sclerosis: study protocol for a prospective randomized clinical trial

**DOI:** 10.1186/s12883-019-1353-z

**Published:** 2019-06-10

**Authors:** Brian J. Loyd, Annie Fangman, Daniel S. Peterson, Eduard Gappmaier, Michael C. Schubert, Anne Thackery, Lee Dibble

**Affiliations:** 1 Department of Physical Therapy and Athletic Training, University of Utah College of Health, 520 Wakara Way, Salt Lake City, UT 84108 USA; 2 Arizona State University, College of Health Solutions, 550 N. 3rd Street, Phoenix, AZ 85004-0698 USA; 3 Department of Otolaryngology Head and Neck Surgery, John Hopkins University School of Medicine, 601 N. Caroline Street, Baltimore, MD 21287 USA; 4 Phoenix VA Health Care System, 650 Indian School Rd., Phoenix, AZ 85012 USA

**Keywords:** Multiple sclerosis, Vestibular rehabilitation, Gaze stability, Postural stability, Randomized clinical trial

## Abstract

**Background:**

The use of vestibular rehabilitation principles in the management of gaze and postural stability impairments in people with multiple sclerosis (PwMS) has shown promise in pilot work completed in our lab and in a recently published randomized clinical trial (RCT). However, further work is needed to fully quantify the gaze and postural impairments present in people with multiple sclerosis and how they respond to rehabilitation.

**Methods/design:**

The study is a single blind RCT designed to examine the benefit of a gaze and postural stability (GPS) intervention program compared to a standard of care (SOC) rehabilitation program in dizzy and balance impaired PwMS. Outcomes will be collected across the domains of body structure and function, activity, and participation as classified by the World Health Organization International Classification of Functioning, Disability, and Health (ICF). Our primary outcomes are the Dizziness Handicap Inventory (DHI) and the Functional Gait Assessment (FGA). Secondary outcomes include other measures of gaze and postural stability, fatigue, and functional mobility. Participants who are interested and eligible for enrollment will be consented prior to completing a baseline assessment. Following the baseline assessment each participant will be randomized to either the GPS or SOC intervention group and will complete a 6 week treatment period. During the treatment period, both groups will participate in guided exercise 3x/week. Following the treatment period participants will be asked to return for a post-treatment evaluation and again for a follow-up assessment 1 month later. We anticipate enrolling 50 participants.

**Discussion:**

This study will be an innovative RCT that will utilize gaze and postural stability metrics to assess the efficacy of vestibular rehabilitation in PwMS. It will build on previous work by examining measures across the ICF and improve the current evidence base for treating PwMS.

**Trial registration:**

ClinicalTrials.gov, May 29th 2018, NCT03521557.

## Background

Multiple Sclerosis is a progressive neurologic disease with a poorly defined etiology that has been linked to genetic, nutritional, infectious, and environmental factors [1]. The study of the disease is further confounded by the varying levels of progression that are categorized into four typical designations based on histologic and clinical presentation: Relapsing Remitting (RRMS), Secondary Relapsing (SRMS), Secondary Progressive (SPMS), and Primary Progressive (PPMS) [1]. Across this spectrum of disease severity the prevalence of MS has been reported to range from 83 to 146/100,000 in North America and Europe, making it the most commonly occurring chronic inflammatory disorder of the central nervous system [1].

Rehabilitative treatment for people living with Multiple Sclerosis (MS) typically includes cardiovascular, strength, and balance training. A large number of clinical trials have demonstrated the benefits of these exercise interventions [2–4]. However, due to the variable presentation in MS and the wide range of symptoms, no single intervention approach has been identified as the gold standard. Therefore, treatment is typically individualized depending upon the individual patient’s signs and symptoms. Among the many presentations common in people with MS are complaints of dizziness, unsteadiness, and poor balance. While these symptoms may have contributions from altered muscle performance, dysfunctions of the vestibular system are a real possibility and often overlooked [5]. In fact, these symptoms have been reported to occur in between 30 and 59% of people living with MS. [6, 7] Unfortunately, until relatively recently, studies have not been designed to examine the benefits of vestibular focused exercises to improve outcomes in people with MS. Furthermore, because of the sparsity of studies, the optimum dosing, exercise type, and duration of vestibular rehabilitation in MS remains unknown.

Recently published studies have taken critical first steps in exploring the use of vestibular based rehabilitation in patients with MS who have complaints of dizziness and poor balance [8, 9]. These studies demonstrated significant improvements in outcomes including patient reported dizziness and scores of the sensory organization test following participation in a vestibular based rehabilitation protocol. To expand on this work, we have designed the current study to examine the use of a vestibular rehabilitation protocol to improve self-reports of dizziness handicap, dynamic stability during gait, as well as gaze and postural stability outcomes. The measures used in this study were chosen to reflect domains of disablement from across the spectrum of the World Health Organization International Classification of Functioning, Disability, and Health (ICF) [10]. Specifically, the outcomes in the domain of body structure and function are measures of the vestibulo-ocular reflex (gaze stability) and the vestibulo-spinal reflex (postural stability). The Activity domain is measured by examining each participant’s ability to stabilize vision during movement and maintain postural stability during various tasks. Participation will be examined using questionnaires pertaining to limitations during life situations as a result of poor gaze and/or postural stability.

The purpose of this manuscript is to summarize the protocol (study design, participants, outcome measures, intervention details, and planned statistical analysis) to be used in a prospective randomized clinical trial (RCT). The objectives of this RCT will be to (1) characterize baseline gaze and postural stability limitations across the domains of the ICF in individuals with MS, and (2) to systematically test the efficacy of a vestibular rehabilitation approach at improving gaze and postural stability in individuals with MS compared to a standard of care intervention.

## Methods/design

**Fig. 1:**
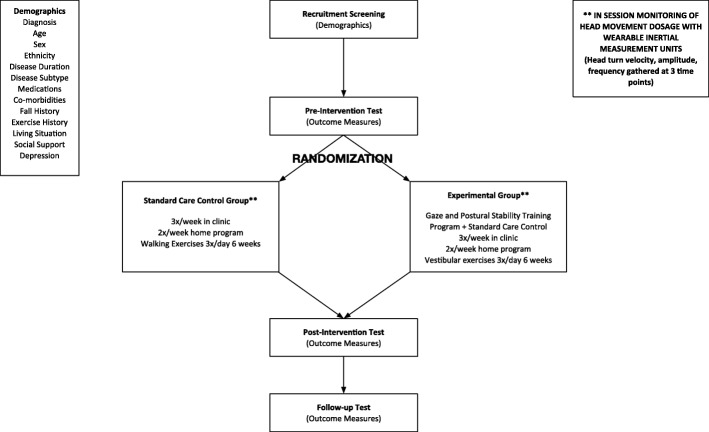
Study flowchart demonstrating the study procedures from screening through study completion

Study design: The proposed project is designed as a single blind RCT. Participants in the trial will be tested at baseline, 6-weeks (post-intervention), and 10-weeks (follow-up). The intervention period lasts for a total of 6 weeks over which participants in both control and intervention groups participate in 18 visits of guided treatment at a frequency of 3x/week. (Fig. 1).

Participants/enrollment: Recruitment will occur through collaborative university and community neurologist clinics, university physical therapy clinics, and through community advertising including advertising through the National Multiple Sclerosis Society. Included participants will be between the ages of 20 and 75, have neurologist diagnosed MS, able to tolerate up to 5 min of continuous head movement, and have dizziness or impaired balance determined by having either experienced ≥2 falls in the last year, a Dizziness Handicap Inventory score > 0, an Activities Balance Confidence scale score < 80%, or a Dynamic Gait Index score < 19. Participants will be excluded if they have neurologic diagnosis other than MS, the presence of peripheral vestibular pathology (i.e., BPPV, hypofunction, or Meniere’s disease), have experienced an exacerbation in MS symptoms within the last 8 weeks, or have an orthopedic, cardiovascular, or other comorbidity that limits exercise participation.

### Power/sample size calculation

Using data from: 1) our pilot research on gaze and postural stability in persons with MS [8] and 2) spatial and temporal measures from previous vestibular research examining the response to task specific training [11–14], we estimated an effect size of d = 0.25 for the DHI with the correlation between baseline and follow-up measurements as 0.50. Twenty participants in each arm provides us with greater than 80% power and 2-sided α = 0.05 to detect a difference between groups on the DHI. We will enroll 50 individuals with MS from various sources to ensure adequate power to examine both DHI and FGA.

### Participants

Participants will be screened in-person or via telephone call for qualification in the study. If study inclusion is met, demographic information and disease history will be collected via interview at the baseline assessment. Following baseline testing participants will be randomized to receive either the standard of care (SOC) or gaze and postural stability (GPS) intervention. Randomization will be performed and delivered to the treatment team by an unblinded research coordinator, while all outcome assessors will remain blind to group assignment. All standardized outcome measures will be collected at each testing timepoint (baseline, post-intervention, follow-up). Intervention protocols for standard of care and gaze and postural stability interventions, as well as all outcome measures are described below.

### Confirmation of central vestibular pathology

The absence of PNS mediated vestibular dysfunction and the presence of CNS mediated vestibular dysfunction will be confirmed by a systematic process. First, we will conduct a focused history including signs and symptoms as well as treatment for *a prior* peripheral vestibular pathology [15]. Second, we will perform clinical testing to rule out peripheral causes of vestibular symptoms (Dix Hallpike and Roll Tests for vertical and horizontal canal BPPV respectively; clinical head impulse testing to test for unilateral or bilateral hypofunction; observation of the presence or absence of direction fixed spontaneous nystagmus with fixation removed). Third, we will confirm the presence of CNS mediated oculomotor deficits (saccadic smooth pursuit, dysmetric saccades, abnormal vestibular ocular reflex [VOR] cancellation) and a perverted head shake nystagmus (fixation removed) [16]. Lastly, we will document the presence or absence of VOR gain deficits and saccadic corrections via head impulse testing that did not follow a pattern consistent with peripheral vestibular pathology [17].

### Gaze and postural stability (experimental) intervention

The GPS intervention is specifically designed to focus on gradually increasing difficulty of gaze and postural stability exercises. The protocol frequency, duration and components were based off of previous intervention trials conducted on peripheral pathology [9, 11–13, 18], as well as, pilot work performed on PwMS [8]. For specific detail regarding the treatment protocol and progression refer to Table 1.

**Table 1:** Specific treatment and progression for experimental group receiving gaze and postural stability (GPS) intervention

Week	Gaze and Postural Stability (GPS) Group Intervention
1	Gaze stability: aVOR × 1 far target, Performed at 2 Hz (metronome paced) × 2 min × 5 reps. Postural stability: standing with static BOS, static COM, static head positions out of neutral (looking, up, down, right, left)
2	Gaze stability: aVOR × 1 near and far targets, 2 targets. Performed at 2 Hz (metronome paced) × 2 min × 5 reps. Postural stability: standing with static BOS, dynamic COM, head rotations (looking, up, down, right, left)
3	Gaze stability: aVOR × 1 near and far targets, aVOR × 2, 2 targets, Performed at 2 Hz (metronome paced) × 2 min × 5 reps. Postural stability: standing / walking compliant surface, static head positions out of neutral (looking, up, down, right, left)
4	Gaze stability: aVOR × 1 near and far targets, aVOR × 2, 2 targets. Performed at 2 Hz (metronome paced) × 2 min × 5 reps. Postural stability: standing / walking compliant surface, head rotations (looking, up, down, right, left)
5	Gaze stability: aVOR × 1 while walking, aVOR × 2 while standing. Performed at 2 Hz (metronome paced) × 2 min × 5 reps. Postural stability: standing / walking, eyes open / closed, head rotations (looking, up, down, right, left)
6	Gaze stability: aVOR × 1 while walking, aVOR × 2 while standing, 2 targets, imaginary target. Performed at 2 Hz (metronome paced) × 2 min × 5 reps. Postural stability: standing / walking, eyes open / closed, head rotations (looking, up, down, right, left)

Progression of exercises will only occur if participants are able to successfully complete the current weeks exercises. aVOR ×1 = horizontal / vertical head motions while maintaining focus on a stationary visual target; Far target = target at 3 m; Near target = target at 1 m; 2 targets = Participant first moves eyes to a target and while maintaining focus on target, moves head to face target; Head rotations = Participant rotates head side to side or up and down; Static base of support (BOS) = feet in place; Static Center of Mass (COM) = stationary body; Dynamic COM = moving body; Dynamic BOS = moving BOS, such as in walking

### Standard of care intervention

The SOC intervention is designed to focus on improving endurance and lower extremity muscular strength. The protocol was based off of documented evidence from previous intervention trials with PwMS [3, 4], as well as, recent evidence from experimental and meta-analytic studies documenting the benefits of exercise (aerobic and resistance) to improve walking ability in people with MS [19–21]. For specific detail regarding the treatment protocol and progression please refer to Table 2.

**Table 2:** Specific treatment and progression for standard care exercise group receiving aerobic and resistance intervention

Week	Standard Care Exercise Group Intervention
1–6	Aerobic Exercise at a moderate exercise intensity (13 on a 20 point Borg Scale) × 30 min^a^; 3x/week Resistance exercise: Lower extremity leg press / concentric at 50% 1RM^b^ × 3 sets × 20 repetitions; 3x/week Lower extremity heel raises / concentric at 50% 1RM** × 3 sets × 20 repetitions; 3x/week

^a^Aerobic Exercise will be performed on a seated NuStep Upper Extremity/Lower Extremity Ergometer. This mode of exercise is chosen because it has been shown to elicit sufficient aerobic challenge while at the same time minimizing postural/vestibular demands because of the sitting position and back support

^b^Lower extremity resistance training will be performed on a seated leg press (Tuffstuff, Pomona, CA). This mode of exercise is chosen because it has been shown to strengthen lower extremity extensors relevant for gait while at the same time minimizing postural / vestibular demands because of the sitting position and back support

### Demographic and anthropometric information

During baseline testing the participants will complete a patient demographics form, which will provide information regarding patient medical history, comorbidities, medications, and personal factors (e.g., history of falls, exercise status). They will also complete information related to the medical history such as the sequence of symptoms that lead to their diagnosis of specific Multiple Sclerosis (MS). We will also record questions regarding their current management of MS symptoms, such as medications, physical therapy, and other treatments.

### Measures of body structure and function

*Video Head Impulse Test (vHIT)* is a measure of gaze stability indicated by the strength of the vestibulo-ocular reflex (VOR) in each of the six semicircular canals. vHIT testing will be performed using the Otometrics ICS impulse system (Natus Medical Inc.). This system quantifies VOR function by calculating the ratio between eye and head velocity, known as VOR gain during low amplitude, moderate velocity, and high acceleration head impulses performed by the examiner. Higher gains indicate better performance with healthy control gain values provided by the manufacturer (VOR gain typically > 0.8 are normal) [22, 23]. Additionally, data regarding the positional error of the eye in relation to the head will be calculated as will various metrics of saccade behavior (i.e. latency, amplitude, and frequency) [24–26]. Testing of VOR function with vHIT is more reliable than clinical non-instrumented head impulse testing and has been used extensively in assessing vestibular function in a variety of diagnoses [27]. Additionally, vHIT testing is validated in PwMS to have significantly more pathological VOR gains compared to healthy controls [28](Table 3).

**Table 3:** Outcome measures utilized in study showing their collective representation of ICF domains

ICF Disablement Construct	Outcome Variable
Body Structure and Function	video Head Impulse Test (vHIT)
	Reactive Stepping
Activity	Dynamic Visual Acuity (DVA)
	Mini-BESTest
	Functional Gait Assessment (FGA)
Participation	Dizziness Handicap Inventory (DHI)
	Activities-Specific Balance Confidence Scale (ABC)
	VAS of Global Dizziness and Balance

*Reactive stepping* is the response to a random perturbation or release causing a quick, highly integrated postural response to recover equilibrium and functions as a measure of postural stability and the VSR [29]. In this study reactive stepping will be performed using a backwards tether release paradigm, during which participants will be asked to lean backward against a force detecting tether until in a range of 8–12% of body weight. When in this range, the tether will randomly release and measures of the latency of the first step, length of the step, and the margin of stability will be captured using 3D motion capture. Previous research has demonstrated that PwMS had larger center of mass displacements and step latencies than healthy controls during a similar corrective stepping task and the magnitude of these deficits significantly correlated to increased severity on clinical outcome measures [29, 30](Table 3).

### Measures of activity

*Dynamic Visual Acuity (DVA)* is a functional assay of the VOR and measures visual acuity during self-generated head rotation. DVA is measured using a head worn accelerometer and a tablet computer (Table 3).

*The Mini BESTest* measures postural stability performance during static, dynamic, and reactive balance activities designed to target 6 different balance control systems. The Mini BESTest has high content validity with gold standard measures of balance deficits such as Berg Balance Scale, Clinical Test of Sensory Integration in, Balance, Dynamic Gait Index, and Timed Up and Go (TUG) test [31]. Studies comparing the Berg Balance Scale to the Mini BESTest, found that the Mini BESTest had a lower ceiling effect and higher values on responsiveness tests, suggesting it may be more sensitive to detecting changes in balance in PwMS who display minimal walking disability [32] (Table 3).

*The Functional Gait Assessment (FGA)*(Primary Outcome) is a commonly used 10 item assessment of dynamic postural stability consisting of a variety of ambulatory tasks including normal walking, walking with head turns, stepping over obstacle, stopping and turning, walking with a narrow base of support, and ascending/descending stairs, among others. The FGA has excellent concurrent validity when compared to the Berg Balance Scale, timed up and go to TUG, and Activities-specific Balance Confidence Scale in many populations who are known to have balance deficits including Multiple Sclerosis, Parkinson’s disease, older adults, vestibular disorders, and stroke [33–35] (Table 3).

### Measures of participation

The *Dizziness Handicap Inventory (DHI)* (Primary Outcome) is a 25 item self-reported questionnaire chosen to examine disability related to gaze instability that evaluates individual perceptions of the impact of dizziness and/or unsteadiness on functional and participation level activities. The questionnaire requires participants to determine if an activity/event never, sometimes, or always causes an increase in dizziness or unsteadiness. Total scores range from 0 to 100, with higher values indicating greater dizziness related disability [36, 37]. The DHI has adequate to excellent correlation with the Berg Balance Scale and the Dynamic Gait Index in PwMS [38, 39] (Table 3).

*Activities-Specific Balance Confidence Scale (ABC)* is a 16 item self-reported measure of postural stability that quantifies the individual’s confidence and/or self-efficacy in performing various dynamic or static activities. Questions require participants to rate their confidence in performing each activity without a loss of balance or a fall from 0 to 100%, with 100% indicating total confidence. The ABC has been validated in PwMS finding concurrent convergent validity was moderate to good (0.50 to − 0.75) with the highest correlation for the 12 item MS walking scale [40, 41] (Table 3).

*Visual Analog Scale of Global Dizziness and Balance* is an analog scale used to rate global intensity of dizziness and balance over the last 48 h. The individual will mark a vertical oriented line on a 10 cm line to indicate the amount of dizziness or unsteadiness they are feeling. The start of the line indicates no symptoms and the end of the 10 cm line indicates the most dizziness or unsteadiness they can experience. Similar scales have been used to measure global pain ratings [42, 43], to quantify visual vertigo [44], and rate the severity of dizziness and unsteadiness [45, 46] (Table 3).

### Controls for threats to internal validity

Additionally, the following measures will be collected as they could have an influence on the dependent measures, but are not considered primary or secondary outcome measures.

*Expanded Disability Status Scale (EDSS)* The EDSS is a valid and reliable indicator of disability used by referring medical providers in PwMS. While we do not expect the EDSS to change as a result of our intervention, these scores are important to characterize the sample of participants and may be important co-variates in our statistical analysis. EDSS scores will be gathered by a trained rater [47].

*Six Minute Walk Test (6MWT)* The distance walked during the 6MWT is a valid and reliable measure of locomotor ability in populations with a variety of chronic diseases including MS. [48] Higher values reflect greater ability. To perform this test, participants will circle a 25-m course continually for 6 min according to the standardized protocol described by the American Thoracic Society [49]. We have previously used this measure with PwMS to examine ambulatory ability using in-clinic and with wearable monitors which measured community ambulatory activity [50, 51].

*Modified Fatigue Impact Scale (MFIS)* The MFIS contains 5 statements that describe how fatigue may impact an individual with MS during the previous 4 weeks. Each item is rated on a 5-point ordinal scale; total scores range from 0 to 20, and lower scores indicating less fatigue. The MFIS has been validated previously as a measure of fatigue in individuals with MS. [52]

*Fatigue Severity Scale (FSS)* The FSS is a 9-item questionnaire commonly used in examination of fatigue in Multiple Sclerosis [53]. It uses a 7 point Likert scale in which users are asked to rate how strongly they agree with a statement pertaining to how fatigue has influenced their life in the last week. Scores range from 9 (lowest fatigue) to 63 (highest fatigue).

### Measures of intervention integrity and compliance

In order to quantify the dosage and progression of the head and trunk movement exercises performed during the intervention period and to confirm that the groups differed in this regard, we will utilize wearable inertial measurement units (IMU) during treatment and track progression with daily notes and participant recorded home exercise logs. At three points over the course of treatment the treatment period (approximately treatment 1, treatment 9, and treatment 18) participants will wear a suite of three IMU sensors placed on the forehead, sternum, and low back. (APDM inc. Opal) While wearing the sensors, the participants will complete their daily exercise routine and data regarding the direction, amplitude, frequency, and velocity of head and trunk turns will be captured. Concurrently, the research staff delivering treatment will track exercises performed, duration, intensity. This will allow us to 1) track progression of vestibulo-ocular reflex exercises in the GPS group, 2) compare the overall magnitude of head movement during treatment between the GPS and SOC groups, and 3) track the progression of aerobic and resistance training exercises in both groups.

Daily notes and home exercise logs will be collected for both groups to determine daily progression of all exercises and to measure compliance to the treatment protocol and home-based exercise prescription. Measures of progression and compliance may serve as important modifying variables in the final analysis.

### Statistical analysis plan

All data will be initially analyzed to determine if assumptions for parametric analysis are met. Decisions regarding appropriate statistical tests will be made based on the normalcy of the data. Corrections for increased type 1 error risk will be performed separately for the data within each specific aim. Post-hoc power and effect size will be calculated for all tests to inform the sample sizes of future studies.

Analyses of longitudinal outcomes will be performed using mixed effects models in which results are robust to missing data as long as the pattern of missingness conforms to the missing at random (MAR) assumption [54]. If more than 10% of follow-up measurements are missing for primary or any of the main secondary outcomes at any of the follow-up visits, sensitivity analyses will be performed after applying multiple imputation to impute missing data under models incorporating predictors of missingness and/or the outcome variables [55].

For the primary outcomes (DHI and FGA) we will apply a linear mixed model [55] to compare mean scores at the post-test and follow-up assessments between the GPS and SOC groups after controlling for baseline scores. Statistical inferences will be performed using restricted maximum likelihood estimation under an unstructured covariance model to account for serial correlations among repeated measurements. The fixed effects terms will include the baseline scores, follow-up visit (as a categorical variable), treatment assignment, and the interaction between follow-up visit and treatment assignment. For secondary outcomes, similar mixed effects analyses will be applied to evaluate the effects of the GPS intervention compared to SOC on the ABC, Mini-BESTest, DVA, vHIT, and reactive postural response tests, adjusting in each case for initial levels of the dependent variable. Monitoring for safety will be conducted throughout the study, investigating all possible unanticipated safety events.

## Discussion

Dizziness and falls are among the most debilitating symptoms reported by people with MS. [5] While a number of studies have attempted to improve balance in people with MS [56, 57] it was not until recently that management of balance and dizziness have been approached using a vestibular rehabilitation perspective [8, 9]. To further explore this innovative rehabilitation approach, we have designed this intervention trial to examine the use of vestibular rehabilitation using a randomized controlled trial design. Outcomes being explored have been categorized as either relating to gaze (dizziness) or postural (balance) stability. Furthermore, we have made it a goal to explore outcomes across the spectrum of disability as defined by the WHO International Classification of Functioning, Disability, and Health. This includes outcomes from the domains of Body Structure and Function, Activity, and Participation related to both gaze and postural stability. The results from this study will significantly add to the literature surrounding the impact and recovery of dizziness and balance impairment in people with MS and ultimately lead to improved rehabilitative management for these people.

## Data Availability

Not Applicable, data is still being collected and all data previously collected will remain blinded until completion of the study.

## Abbreviations

6MWT: Six minute walk test
ABC: Activities balance confidence scale
CNS: Central nervous system
DHI: Dizziness handicap inventory
DVA: Dynamic visual acuity
EDSS: Expanded disability status scale
FGA: Functional gait assessment
FSS: Fatigue severity scale
GPS: Gaze and postural stability
IMU: Inertial measurement units
MAR: Missing at random
MFIS: Modified fatigue impact scale
MS: Multiple sclerosis
PNS: Peripheral nervous system
PPMS: Primary progressive multiple sclerosis
PwMS: Persons with multiple sclerosis
RCT: Randomized clinical trial
RRMS: Relapsing remitting multiple sclerosis
SOC: Standard of care
SPMS: Secondary progressive multiple sclerosis
SRMS: Secondary relapsing multiple sclerosis
vHIT: Video head impulse test
VOR: Vestibular ocular reflex
VSR: Vestibulospinal reflex
WHO ICF: World health organization international classification of functioning, disability, and health
